# Supplementary material for: The Antiasthma Medication Ciclesonide Suppresses Breast Cancer Stem Cells through Inhibition of the Glucocorticoid Receptor Signaling-Dependent YAP Pathway
Source: Molecules. 2020 Dec 19;25(24):6028. doi: 10.3390/molecules25246028 (PMC7766992; doi:10.3390/molecules25246028)
Supplement: Supplementary file 1 [file molecules-25-06028-s001.pdf]

Supplementary Table S1. Specific Real-time RT-qPCR primer sequences containing human *CD44, Nanog, Sox2, Oxt4, GR, YAP, CTGF, CYR61, and β-actin* genes

Genes	Primers
CD44	Forward: 5′-AGAAGGTGTGGGCAGAAGAA-3′<br>Reverse: 5′-AAATGCACCATTTCCTGAGA-3′
Nanog	Forward: 5′-ATGCCTCACACGGAGACTGT-3′<br>Reverse: 5′-AAGTGGGTTGTTTGCCTTTG-3′
Sox2	Forward : 5′-TTGCTGCCTCTTTAAGACTAGGA-3′<br>Reverse : 5′-CTGGGGCTCAAACTTCTCTC-3′
Oct4	Forward: 5′-AGCAAAACCCGGAGGAGT-3′<br>Reverse: 5′-CCACATCGGCCTGTGTATATC-3′
GR	Forward:5′-GAAGGAAACTCCAGCCAGAA-3′<br>Reverse:5′-CAGCTAACATCTCGGGGAAT-3′
YAP	Forward:5′- GAACCCCAGATGACTTCCTG-3′<br>Reverse:5′- 5′-CTCCTTCCAGTGTTCCAAGG-3′
CTGF	Forward: 5′-CCAATGACAACGCCTCCTG-3′<br>Reverse: 5′-TGGTGCAGCCAGAAAGCTC-3′5′-
CYR61	Forward: 5′-AGCCTCGCATCCTATACAACC3′<br>Reverse: 5′-TTCTTTCACAAGGCGGCACTC3′
β-actin	Forward: 5′-TGTTACCAACTGGGACGACA-3′<br>Reverse : 5′-GGGGTGTTGAAGGTCTCAAA-3′

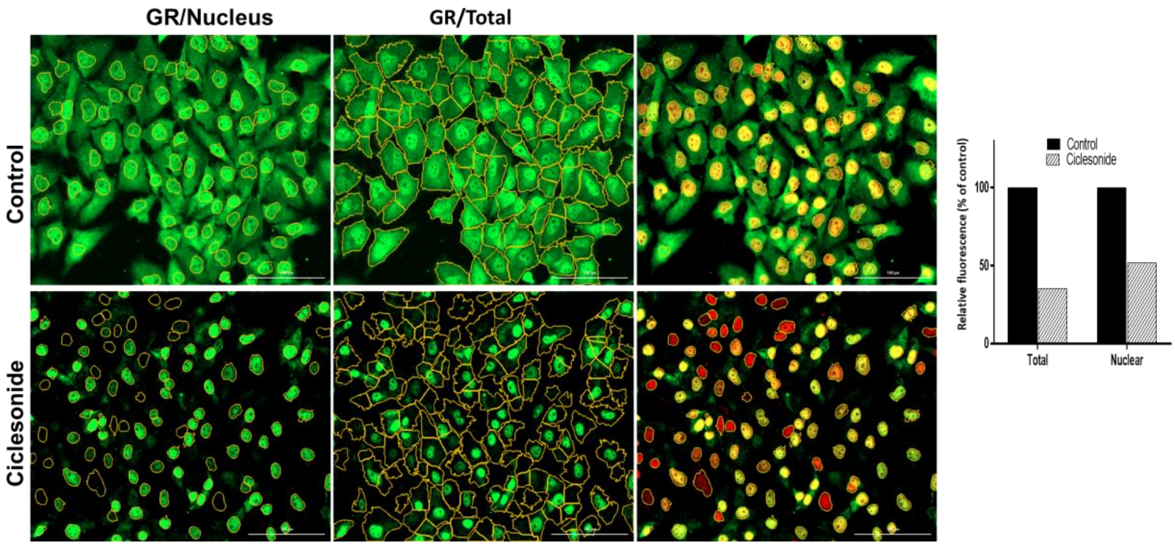

Supplementary Figure S1. Ciclesonide reduced the levels of cytosolic and nuclear GR protein (green) in MDA-MB-231 cells, as evidenced by immunofluorescence. Nuclei were stained with DAPI (red), and GR was labeled with an anti-GR antibody (green). Magnification, x100. Total and nuclear fluorescence with/without ciclesonide were determined by using Gen5 cell imaging program of Lionheart FX machine.

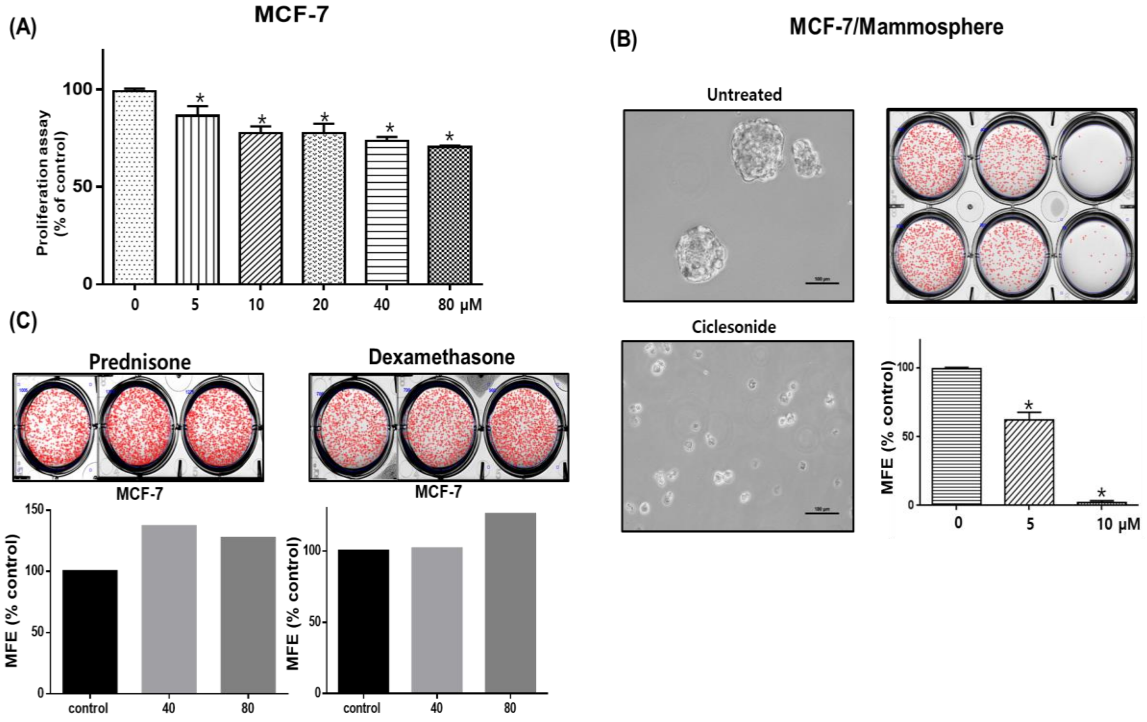

Supplementary Figure S2. Ciclesonide reduced the proliferation of MCF-7 cells. (A) MCF-7 cells were cultured in a plate with the ciclesonide. The growth of cancer cells was assessed with MTS reagent. (B) Mammospheres were cultured for 7 days in MammoCult medium. Treatment with ciclesonide (5 and 10 μM) reduced mammosphere formation to 5% under control conditions. *p<0.05 vs. the control. (C) Treatment with prednisone or dexamethasone (40 and 80 μM) did not reduce the MFE.
